# Research productivity of staff in NHS mental health trusts: comparison using the Leiden method

**DOI:** 10.1192/pb.bp.113.042630

**Published:** 2014-02

**Authors:** Alex J. Mitchell, John Gill

**Affiliations:** 1 Leicestershire Partnership NHS Trust and University of Leicester, UK; 2 University of Leicester, UK

## Abstract

**Aims and method** To examine research productivity of staff working across 57 National Health Service (NHS) mental health trusts in England. We examined research productivity between 2010 and 2012, including funded portfolio studies and all research (funded and unfunded).

**Results** Across 57 trusts there were 1297 National Institute for Health Research (NIHR) studies in 2011/2012, involving 46 140 participants and in the same year staff in these trusts published 1334 articles (an average of only 23.4 per trust per annum). After correcting for trust size and budget, the South London and Maudsley NHS Foundation Trust was the most productive. In terms of funded portfolio studies, Manchester Mental Health and Social Care Trust as well as South London and Maudsley NHS Foundation Trust, Oxford Health NHS Foundation Trust and Cambridgeshire and Peterborough NHS Foundation Trust had the strongest performance in 2011/2012.

**Clinical implications** Trusts should aim to capitalise on valuable staff resources and expertise and better support and encourage research in the NHS to help improve clinical services.

The UK has a strong track record in scientific research, and in particular in medical research. UK researchers published 123 594 articles in 2010.^1^ Of the top five most productive research nations (USA, China, UK, Japan, Germany), UK researchers generate more articles per researcher, more citations per researcher, and more usage per article authored as measured by global downloads of articles arising from UK authors. Although about 80% of research arises in higher education institutes (largely universities), 20% arises elsewhere. One of the UK's largest sources of scientific research is research conducted by National Health Service (NHS) clinicians and academics with honorary NHS contracts. A Wellcome Trust report found that the NHS in England supported over 13 000 publications a year during the 1990s, increasing to about 20 000 per year at the current time.^2^ Mental health accounts for about 5% of that output.

The first NHS research and development strategy was published in 1991^3^ and in 1994 the Culyer Report made recommendations to increase the accountability and transparency of research funding in the NHS.^4^ In 2006, the *Best Research for Best Health*^5^ strategy document led to the creation of the National Institute for Health Research (NIHR). The NIHR had a budget of £992 million in 2010/2011 and have announced the following annual programme budgets totalling £242 million: research for patient benefit (£25 million), programme grants (£75 million), invention for innovation (£13 million), efficiency and mechanisms evaluation programme (£15 million), health service research programmes (£5 million), health technology assessment programme (£88 million), public health research programme (£10 million) and service delivery organisation (£11 million). The Department of Health's research and development budget for the NHS for 2008–2009 was £792 million and £1025 million in 2010–2011.^6^ An additional £760 million of government funding is administered through the Medical Research Council (MRC). The MRC and NIHR are independent bodies. Studies conducted with the assistance of NIHR are often considered of special significance for mental health trusts because trusts are allowed to hold this funding directly. In addition to spending on front-line research as per study grants, trusts typically use this money to fund research assistants, research administrators and clinical studies officers who promote and facilitate research. However, the majority of research in the NHS is not portfolio (NIHR or MRC) research; indeed, about a quarter of all research output is not externally funded. Unfunded research is typically conducted by motivated staff that usually fit research into busy clinical jobs with little or no central support.

Recently, it has become possible to compare institutions based on research output. Scholarly productivity is often measured by the number of articles published in peer-reviewed journals or proxy measures such as the number of participants enrolled in research or the number of registered funded studies.^7^ A wide variety of methods are available to compare institutions (e.g. http://en.wikipedia.org/wiki/College_and_university_rankings). It is generally advisable to account for the size of institutions. One of the most well-known methods is the Leiden ranking. This was developed by the Centre for Science and Technology Studies at Leiden University which provides a ranking of the top 500 universities according to the number of Web of Science-indexed publications per year, after accounting for institutional size.^8^ The full Leiden method takes into account different impact indicators, different counting methods, weighting of collaborations and non-English language publications (see www.leidenranking.com/methodology.aspx). Additional ranking lists are available according to various bibliometric normalisation and impact indicators, including the number of publications, citations per publication, and field-averaged impact per publication (see www.leidenranking.com/ranking). The Leiden Ranking is fundamentally based on publications in Thomson Reuters' Web of Science database in a recent 5-year period. Only publications in the sciences and the social sciences are included. Furthermore, only publications of the Web of Science document types article, letter and review are considered in the Leiden ranking (for further details see www.leidenranking.com/methodology.aspx). Given the information available at the current time, we chose the Leiden method. We aimed to replicate the Leiden method to compare mental health trusts in England, without weighting of collaborations. In this analysis, collaborations were counted for each listed institution.

## Method

We examined research productivity in 57 current mental health trusts in England between 2010 and 2012. For funded portfolio studies we used two measures of research productivity: number of NIHR studies (2010/2011 and 2011/2012)^9^ and number of NIHR recruited participants (2010/2011 and 2011/2012).^9^ Given the wide variation in size of mental health trusts, we made two adjustments for trust size in order to calculate research efficiency. We divided productivity (as above) by either the number of employed staff (usually given as full-time equivalents and available online or in annual reports) or the trust budget (usually given as turnover in 2011/2012 available online or in annual reports). These two outputs and two adjustments allowed the trusts to be measured in four domains and ranked accordingly. All four rankings were combined by calculating the sum of each individual ranking position across four domains (studies/staff ranking + participants/staff ranking + studies/budget ranking + participants/budget ranking). This combined performance index (combination score) was based on rankings not raw scores in order to give equal emphasis to each domain and adjust for outlier performance. The combined performance index generated an overall ranking position, although individual performance for each domain is also shown (online Table DS1). Unfunded studies are difficult to quantify as there is no register of studies or participants and therefore we used the Leiden method to examine published peer-reviewed research on Web of Science over the past year (2011/2012) as well as over the past 5 years (2007–2012). As most of these studies did not cite funding sources we considered them to be evidence of unfunded research publications. Web of Science contains about 50 million records and is considered to be the largest accessible citation database. The change in productivity judged by the difference in mean published research over 5 years *v*. published research over the past year gives an indication of the trend in recent productivity for any given trust.

## Results

We located data on 57 mental health trusts. The mean trust size by staffing was 3518 (range 527–8800) and the mean budget was £162.8 million (range £30–410 million). Across 57 trusts, in 2011/2012 staff registered 1297 NIHR studies (an average of 22.8 per trust), involving 46 140 participants (an average of 809 per trust) and in the same year published 1334 publications (an average of 23.4 per trust). A quadrant analysis of relatively productive trusts according to NIHR portfolio and published papers (funded and unfunded) is shown in Fig. 1.

### Funded NIHR ‘portfolio’ research by trust size

Output by trust size can be considered a measure of research efficiency. Once trust size was accounted for by staffing numbers, the top five trusts for funded NIHR portfolio research were: 1. Manchester Mental Health and Social Care Trust; 2. South London and Maudsley NHS Foundation Trust; 3. Oxford Health NHS Foundation Trust; 4. Cambridgeshire and Peterborough NHS Foundation Trust; and 5. Lincolnshire Partnership NHS Foundation Trust. The bottom five trusts for funded NIHR portfolio research were: 53. Dorset Healthcare University NHS Foundation Trust; 54. Worcestershire Mental Health Partnership NHS Trust; 55. Rotherham, Doncaster and South Humber NHS Foundation Trust; 56. Calderstones Partnership NHS Foundation Trust; and 57. Oxfordshire Learning Disability NHS Trust. The trusts showing most growth compared with 2010/2011 were: 1. East London NHS Foundation Trust; 2. Derbyshire Healthcare NHS Foundation Trust; and 3. North Staffordshire Combined Healthcare NHS Trust.

Sub-analysis of the top five trusts for research recruitment (number of NIHR participants/staff per capita) reveals the following: 1. East London NHS Foundation Trust; 2. Manchester Mental Health and Social Care Trust; 3. South London and Maudsley NHS Foundation Trust; 4. Camden and Islington NHS Foundation Trust; and 5. Oxford Health NHS Foundation Trust. The top five trusts for funded research value (number of NIHR studies/trust budget) were: 1. Berkshire Healthcare NHS Foundation Trust; 2. Manchester Mental Health and Social Care Trust; 3. Cambridgeshire and Peterborough NHS Foundation Trust; 4. Lincolnshire Partnership NHS Foundation Trust; and 5. South London and Maudsley NHS Foundation Trust.

### Total research output

After accounting for trust size by staffing numbers, the top five most productive trusts in terms of publications

**Fig 1:**
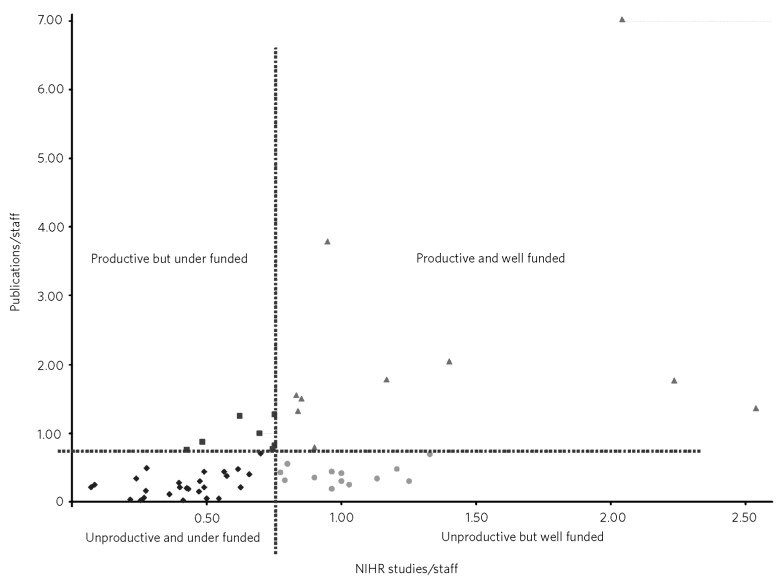
Productive *v*. unproductive mental health trusts (quadrant analysis). Productive means more than average ratio of publications/staff. Unproductive means less than average ratio of publications/staff. Well funded means more than average ratio of funded National Institute for Health Research (NIHR) studies/staff. Under funded means less than average ratio of funded NIHR studies/staff.

were: 1. South London and Maudsley NHS Foundation Trust; 2. Tavistock and Portman NHS Foundation Trust; 3. Cambridgeshire and Peterborough NHS Foundation Trust; 4. Berkshire Healthcare NHS Foundation Trust; and 5. Greater Manchester West Mental Health NHS Foundation Trust. The least productive trusts were: 53. Black Country Partnership NHS Foundation Trust; 54. Suffolk Mental Health Partnership NHS Trust; 55. Worcestershire Mental Health Partnership NHS Trust; 56. Rotherham, Doncaster and South Humber NHS Foundation Trust; and 57. South West Yorkshire Partnership NHS Foundation Trust. The trusts showing most growth in productivity over the past 5 years were: 1. South London and Maudsley NHS Foundation Trust; 2. Tavistock and Portman NHS Foundation Trust; and 3. Cambridgeshire and Peterborough NHS Foundation Trust.

Sub-analysis of total research efficiency (number of published studies/staff per capita) showed that the top five trusts were: 1. South London and Maudsley NHS Foundation Trust; 2. Tavistock and Portman NHS Foundation Trust; 3. Cambridgeshire and Peterborough NHS Foundation Trust; 4. Camden and Islington NHS Foundation Trust; and 5. Berkshire Healthcare NHS Foundation Trust. Similarly, the trusts offering best research value for money were: 1. South London and Maudsley NHS Foundation Trust; 2. Tavistock and Portman NHS Foundation Trust; 3. Cambridgeshire and Peterborough NHS Foundation Trust; 4. Berkshire Healthcare NHS Foundation Trust; and 5. Greater Manchester West Mental Health NHS Foundation Trust.

## Discussion

This is the first comprehensive comparison of research productivity of mental health trusts in England. Productivity of individual staff is known to vary considerably and although some staff in mental health trusts have excellent productivity, overall staff showed relatively low output. Across 57 trusts in 2011/2012 staff registered 1297 NIHR studies, involving 46 140 participants and in the same year published 1334 publications. An average mental health trust employing about 3500 people would register about one NIHR study and publish one peer-reviewed paper every 2 weeks. Although the number of published publications and number of registered NIHR studies was about equal, there was only a modest relationship between the two (adjusted R^2^ = 0.31). This is because many of the publications appeared to come from unfunded non-NIHR work. This also implies that trust portfolio activity is often not being published in peer-reviewed journals either because of a delay in completing the research, difficulty in getting published or a failure to write up the research. It would be interesting to look at the conversion rate of portfolio studies into publication as well as the conversion rate of unfunded studies, but this is beyond the scope of this paper.

### Published research

On a background of relatively low academic output there was much variation with some trusts and many individuals performing strongly. Staff at one trust, the South London and Maudsley NHS Foundation Trust, were more than twice as effective at producing publications than those at the nearest rival, whereas other trusts produced almost no published research in the past 5 years (online Table DS2). Even after correcting for trust size and budget, the South London and Maudsley NHS Foundation Trust was the most productive, producing 7 publications per 100 staff years, followed by Tavistock and Portman NHS Foundation Trust (3 publications per 100 staff years) and then Cambridgeshire and Peterborough NHS Foundation Trust (2 publications per 100 staff years). It is worth noting that the mean number of publications per 100 staff years was 0.73, meaning that on average NHS mental health trusts produce 1 paper per 137 staff years. It would be interesting to refine this further to employed clinicians but these data were unavailable. Of course, most staff would be entirely unproductive in research and probably not encouraged or supported in this regard. The aforementioned productive trusts were not only the most effective trusts at producing research but they also showed the most (absolute) growth in research over the past 5 years. When examined for relative growth, the top performing trusts were Oxford Health NHS Foundation Trust, Sussex Partnership NHS Foundation Trust and Cumbria Partnership NHS Foundation Trust. In fact, annual research output across all mental health trusts has almost doubled over the past 5 years perhaps reflecting success of the NHS research and development strategy or perhaps reflecting wider research involvement in general. Of course trusts with a high research reputation tend to attract highly motivated employees and also offer those employees a high degree of infrastructural support.

### Funded research

We also examined ranking according to funded research in the form of portfolio studies. The trusts which demonstrated particularly strong performance were Manchester Mental Health and Social Care Trust as well as South London and Maudsley NHS Foundation Trust, Oxford Health NHS Foundation Trust and Cambridgeshire & Peterborough NHS Foundation Trust. Several trusts do not do well at producing published research but do appear to have attracted successful NIHR portfolio research. These are shown in the bottom right of the quadrant analysis (Fig. 1). These mental health trusts include Cheshire and Wirral Partnership NHS Foundation Trust, Lincolnshire Partnership NHS Foundation Trust, North Staffordshire Combined Healthcare NHS Trust and Norfolk & Waveney Mental Health NHS Foundation Trust. One interpretation is that these trusts have been historically relatively research inactive but are focusing on funded research programmes that might lead to greater productivity over the next 5 years. However, their investment in research has yet to produce significant output compared with other trusts. Trusts in this quadrant may be advised to help their clinicians convert ongoing studies into published work perhaps by offering local training and perhaps support from academics at universities linked with these trusts. Conversely, a number of trusts have relatively poor performance in portfolio research compared with their performance in unfunded non-portfolio research. These are shown in the top left of the quadrant analysis (Fig. 1). A clear example is Leicestershire Partnership NHS Trust which has the worst ratio of funded portfolio activity to (largely unfunded) published output. In these mental health trusts clinicians were actually productive but probably without infrastructural support and without strong success in gaining external funding. Trusts with relatively high productivity outside of portfolio research should examine which clinicians could be further supported locally. It is likely these trusts are over-reliant on clinicians producing research in their own time and in the limited hours given to supporting professional activities. Examples of research support that trusts could consider include dedicated sessions for research, research advice and expertise, support for research administration and governance, appointment of honorary research assistants, availability of good literature searching facilities and online journal subscriptions and links with departments with statistical or epidemiological expertise. It is interesting to ask what separates research active mental health trusts from relatively research inactive mental health trusts? Clinicians and managers in NHS trusts that are research inactive, probably consider research as a luxury which they are unable to support over and above face-to-face contacts. This may be particularly true in the modern NHS focusing on payment by results and foundation status. Clinicians and managers in trusts that are research active would likely take the view that research is an integral part of the NHS and vital to support innovation and helps improve existing practice. Indeed, 93% of the public want their local NHS to be encouraged or required to support research.^10^ Research is part of the NHS core principles.^11^ It is supported in the NHS constitution as follows:

‘Research is a core part of the NHS. It enables the NHS to improve the current and future health of the people it serves. The NHS will do all it can to ensure that patients, from every part of England, are made aware of research that is of particular relevance to them.’ (p. 53)^12^

In our view, each trust should evaluate its research output, research support and research funding on an annual basis and set up systems that encourage valuable, ethical research.

This analysis of research productivity can only make limited observation of individuals. Further, many staff employed by trusts are not clinicians and would not be expected to engage in research. Nevertheless, it is rare for trusts to achieve more than 3 publications per 100 staff years (equivalent to 100 employees working for 1 year). Indeed, most clinicians are entirely research inactive. This has been previously documented. For example, only 24% of psychology graduates wrote up their research for publication.^13^ Atkinson & el-Guebaly^14^ surveyed 861 members of the Canadian psychiatric association: 8.1% indicated no research activities over the past 2 years, whereas 50 (33.8%) indicated limited commitments to research and 58.1% indicated a major commitment to research activities, and 67.6% of respondents indicated they were mentors to students. Much research in the NHS is unfunded but we were not able to quantify this proportion. Previous findings are that 26%-38% of research is unfunded^15^ and of those conducting research without funds, 60% carry out research in their own time.^16^

Research by staff and clinicians employed by mental health trusts is increasing. As a whole NHS trusts are increasingly recognising the value of research, but while some innovative trusts are actively promoting research, others appear to focus only on financial income related to research. For employees, integration of research and clinical practice is correlated with greater job satisfaction and personal satisfaction.^17,18^ Underperforming trusts should consider whether greater resources are needed to support relevant research. Clinically relevant research and good-quality audit is an important way to improve patient care by developing and testing new innovations, but too often good work does not get published. The NHS is in a key position to lead on clinical research, but research infrastructure and relevant resources are very variable. Many clinicians from a variety of professional backgrounds have the ability to conduct meaningful research but need the support and encouragement of their employers to do so successfully.
